# Prognostic Biomarkers and Precision Psychiatry: A Review of the Available Evidence

**DOI:** 10.3390/biomedicines14030558

**Published:** 2026-02-28

**Authors:** Itziar María Béjar-Botello, Sara Jiménez-Fernández, Gloria Pérez-Guerrero, Blanca Iglesias-Rosado, Luis Gutiérrez-Rojas, Jesús Herrera-Imbroda, Inmaculada Romera

**Affiliations:** 1 Universidad de Málaga, Andalucía Tech, Departamento de Salud Pública y Psiquiatría, Campus de Teatinos s/n, 29071 Malaga, Spain; 2 Department of Psychiatry, University of Granada, 18071 Granada, Spain; 3 Psychiatry and Neurosciences Research Group (CTS-549), Institute of Neurosciences, University of Granada, 18071 Granada, Spain; 4 Child and Adolescent Unit, Psychiatry Service, Virgen de las Nieves University Hospital, 18014 Granada, Spain; 5 IBIMA Plataforma Bionand, Instituto de Investigación Biomédica de Málaga, 29590 Malaga, Spain; 6 Unidad de Gestión Clínica de Salud Mental, Hospital Regional Universitario de Málaga, 29010 Malaga, Spain; 7 Psychiatry Service, Hospital San Cecilio, 18016 Granada, Spain

**Keywords:** precision psychiatry, prognostic biomarkers, psychiatric disorders, neuroimaging, genetics, inflammation, response to treatment, relapse, therapeutic personalization

## Abstract

Precision psychiatry aims to overcome clinical heterogeneity by means of biomarkers that allow predicting the clinical evolution and therapeutic response in psychiatric disorders. This literature review addresses its prognostic role and its potential integration into healthcare practice. The main objective was to compile and synthesize current evidence on prognostic biomarkers in psychiatry, evaluating their usefulness in anticipating clinical evolution, therapeutic response, and risk of relapse. A strategic search was carried out on PubMed, selecting original studies that evaluated blood, genetic, epigenetic, neuroimaging, or electrophysiological biomarkers with prognostic value. We included 30 final studies that met the established inclusion and exclusion criteria and were evaluated according to standardized scales (RoB 2, NOS, AXIS). Inflammatory biomarkers showed potential as clinical modulators. Metabolomic, neuroendocrine, and neurotrophic factors reflected specific biological profiles associated with response to treatment or risk of relapse. Functional connectivity and brain morphometry were useful in the therapeutic prediction and stratification of patients. Finally, genetics and epigenetics are consolidated as tools of sensitivity and pharmacological response. Taken together, the findings reveal specific prognostic utility based on the type of biomarker and the patient’s clinical context. Despite the current methodological limitations and scarce replication of studies, prognostic biomarkers represent a step towards a more personalized psychiatry based on biological mechanisms. The future integration of multimodal models will improve clinical decision-making.

## 1. Introduction

Prognosis and prediction of therapeutic response in psychiatry remain a complex challenge, with some unresolved questions. Precision psychiatry is a novel therapeutic area that is emerging to provide an answer to these unknowns, trying to be able to discern from early stages of the disease which treatment will be appropriate for each patient, thus optimizing interventions and reducing clinical uncertainty [1,2].

This new branch consists of the search for tools capable of making the diagnosis more precise, indicating prognosis, guiding treatment, and response to it. Precision studies aim to establish subtypes of patients using existing and recorded data, thus being able to predict the prognosis and susceptibility to treatment [3]. The subtypes must be transdiagnostic and cover multiple pathologies, thus finding common patterns that integrate biological and clinical data [4,5].

Traditional systems such as the DSM-5 [6] have limitations when it comes to representing the heterogeneity that characterizes psychiatric conditions, as they exclusively contemplate and categorize clinical symptoms [4,5]. Similarly, there is a disconnect between conventional studies and actual clinical practice, as randomized clinical trials and meta-analyses yield very broad results in broad populations without reflecting particularities [7].

On the other hand, two recent phenomena have increased the amount of information available about the natural history of different psychiatric disorders in specific individuals: important advances in their biological understanding and the widespread use of individual clinical records with comprehensive information on each patient [1]. Such information could lay the foundation for a model capable of pointing out complex nonlinear patterns that associate predictors with outcomes. Thus, software could be developed using artificial intelligence and machine learning that effectively standardizes the analysis of *big data* and that transfers this data to routine clinical practice and the real patient [5,7].

All of the above underscores the need to find these base tools mentioned above: biomarkers of prognosis and response to treatment. Biomarkers are defined as measurable and objective indicators of a biological condition or state. Applied to precision psychiatry, they may be able to predict the course of the disease and the effectiveness of treatment [3]. However, its inclusion in practice faces other challenges: low sensitivity and replicability, methodological variability, and the absence of a *diagnostic gold standard* [2,3,5].

When we talk about biomarkers in psychiatry, it is convenient to distinguish diagnostic, prognostic, and treatment-predictive biomarkers. The former have received a lot of attention recently but are limited by the fact that they actually define, instead of a specific diagnosis, a state of severity or disease that is not specific to the disorder. They are sometimes called “trait markers” [8]. The latter refer mainly to those indicators that can indicate a specific state of the disease and guide clinicians on the natural history of the disease, which is why they have also been known as “state markers” [8]. Finally, biomarkers related to treatment response are useful tools to assess adherence or predict drug responses [9,10]. They are generally considered a special type of prognostic biomarker, which could be especially useful in clinical practice.

Despite the optimism that this so-called “precision psychiatry” has been able to generate so far, the truth is that the current evidence available comes mainly from observational studies and small and/or heterogeneous samples, so we must be very cautious in the correct interpretation of its possible practical applications. Therefore, this review aims to synthesize and compile the evidence available in original studies on the existence of prognostic (and treatment-predictive) biomarkers in psychiatry, describing their ability to individualize therapeutic interventions and identify the risk of relapse and clinical course. This research identifies the achievements and the weaknesses present in the field, providing a basis for future research and improvements in clinical practice.

## 2. Methods

### 2.1. Study Design

A semi-systematic review of the literature and a summary of the available evidence are provided in the following subsections.

### 2.2. Inclusion and Exclusion Criteria

The inclusion criteria were based on selecting original studies in the population with psychiatric disorders. These included both experimental studies (clinical trials, intervention trials, etc.) and observational studies (cohorts, case–control, or cross-sectional). The selected psychiatric population includes both adults and children in order to represent both subgroups and the relevance of the results. Exclusion criteria ruled out reviews or meta-analyses, as a primary and direct approach to empirical data is needed. In addition, studies carried out in non-psychiatric areas, studies that did not mention biomarkers in the full text, animal-based studies, and studies on biomarkers that indicated exclusively diagnosis (and therefore no prognostic value) were excluded.

### 2.3. Search Strategy

The search was carried out using the PubMed database, chosen for its extensive catalog in biomedical literature and relevance in the field of biomarkers. To conduct the search, we used terms based on inclusion and exclusion criteria that could cover the major psychiatric disorders and biomarker groups described in the existing literature on precision psychiatry, in the interest of greater specificity and efficiency in data extraction. Finally, the following search strategy was used:

(“precision psychiatry” OR “personalized psychiatry” OR “psychiatric disorder” OR “schizophrenia” OR “bipolar disorder” OR “depression” OR “Mood disorder” OR “PTSD” OR “Anxiety disorder” OR “psychiatric patients” OR “telepsychiatry”)

AND

(“biomarkers” OR “genetic biomarkers” OR “biological markers” OR “neuroimaging biomarkers” OR “inflammatory biomarkers” OR “EEG biomarkers”)

AND

(“prognosis” OR “prognostic value” OR “outcome prediction” OR “functional outcomes” OR “hospital readmission” OR “relapse” OR “recurrence” OR “relapse prevention”).

After the initial search for studies, we performed a backward citation search against reference lists to ensure that no major studies were included in the review.

### 2.4. Selection of Studies

**Figure 1:**
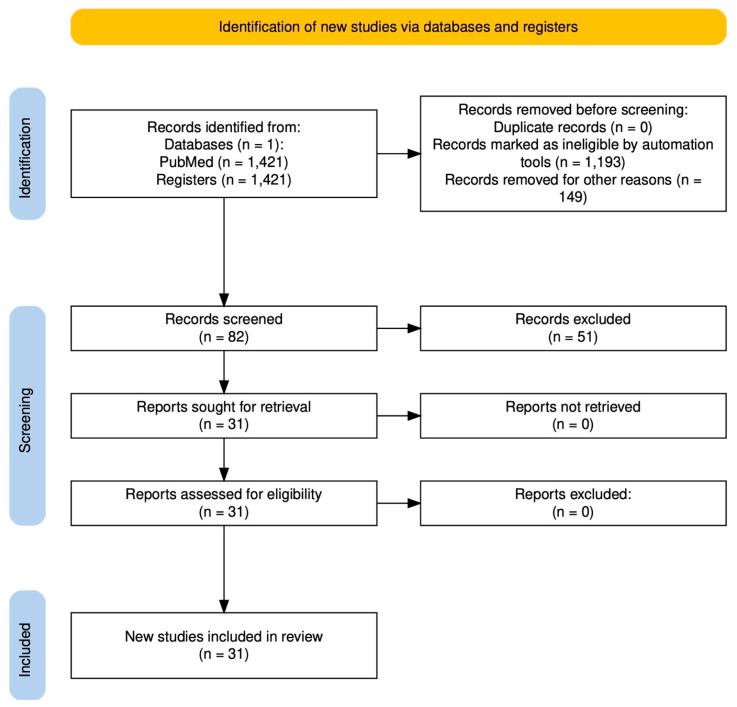
Flowchart.

The studies identified from the search strategy were 1421. Automatic tools based on study type filters removed 1193 items. Duplicate studies totaled 0, while those eliminated for other reasons (not focusing on psychiatry) totaled 149. Rayyan web-based software (Rayyan Systems Inc., Cambridge, MA, USA) was used to review the 82 studies resulting from the search. Manual screening based on exclusion and inclusion criteria led to the selection of 30 studies that met these criteria. The complete flowchart is represented in Figure 1.

### 2.5. Data Extraction

Data extraction is found in Table 1. The author, year, and study design; the characteristics of the population studied and the sample size; the specific biomarkers evaluated; the method of evaluation and its type; level of evidence and risk of bias; and finally, the main results and objectives of the studies have been considered.

### 2.6. Assessment of Quality and Risk of Bias

Various assessment scales were applied manually depending on the design of each study. For randomized clinical trials, RoB 2 (Cochrane Risk of Bias 2) and NOS (Newcastle-Ottawa Scale) were used for observational cohort or case–control studies, and the AXIS Tool was used for cross-sectional studies. In the case of exploratory genetic, proteomic, or neuroimaging studies, in the absence of specific standardized tools, adapted criteria (internal validity, methodological transparency, replicability, and sample size) were applied.

The result of this assessment is reflected in Table 1, classified as low, moderate, or high risk of bias and high, moderate, or low level of evidence. In each entry, this categorization is justified.

## 3. Results

The main findings of the review can be found in Table 1. Of the 30 studies that were finally included for review, 20 were longitudinal observational studies, 3 were cross-sectional studies, and 7 were experimental studies. The cumulative total n of all studies was 7363. In terms of diagnoses, 11 studies referred to the population with schizophrenia or related disorders, 16 to major depression, 2 to bipolar disorder, and 1 to generalized anxiety disorder. Regarding the methodological quality of revised studies, low levels of bias predominated, while high levels prevailed in the evidence. Of the 30 studies analyzed, 15 had a low level of bias, 14 had a moderate level, and 1 had a high level. The articles with the lowest risk of bias were mostly randomized clinical trials, with clear randomization, well-defined control groups, use of objective measurements, and robust statistical analysis.

Regarding the level of evidence, 16 studies presented high, 13 moderate, and 1 low levels. The strongest evidence came from studies with longitudinal follow-up, large samples, cross-validation, and standardized quantitative techniques.

In conclusion, the methodological evaluation offers a significantly robust empirical base, with mostly well-designed studies and no risk of critical bias.

Below are the main groups of biomarkers studied.

### 3.1. Immune Biomarkers

Several studies included in this review agree that inflammatory biomarkers (e.g., IL-6, IL-1β, TNF-α, and CRP) have a significant correlation with the severity of depressive symptoms, specifically in their somatic and neurovegetative dimensions. However, they do not consistently predict overall therapeutic response or the risk of relapse [11].

On the other hand, another study indicates that there are biomarkers related to subjective cognitive impairment. Plasma levels of basic FGF, INF-γ, IL-1β, MCP-1, M-CSF, and SCF were higher in patients with this clinical dimension, while IL-9, RANTES, and PDGF-BB were lower in this group. In addition, high accuracy was found in a predictive model composed of SCF and PDGF-BB combined with the initial PDQ-D (Perceived Deficits Questionnaire for Depression) assessment after antidepressant treatment [12].

In relation to remission in depression, it was found that elevated levels of IL-6 and CRP at the beginning of treatment were related to a lower probability. It was only seen in patients treated with CBT, not those treated with antidepressants [13].

Regarding the transition to psychosis, two proteomic profiling studies identified systemic inflammatory markers (complement pathways, coagulation cascade, or alpha-2-macroglobulin) with moderate predictive capacity in adolescents at high clinical risk [14,15].

Finally, it was observed that high monocyte and basophil counts were related to the risk of psychotic relapse in patients with schizophrenia in remission. In contrast to a high PRL, it behaved as a protective factor [16].

### 3.2. Metabolomic Biomarkers

In response to antidepressant treatment, higher baseline concentrations of phosphatidylcholine C38:1 were found to be associated with a lower reduction in depressive symptoms after treatment, suggesting an association with antidepressant resistance. In contrast, a ratio of hydroxylated sphingomyelins over non-hydroxylated sphingomyelins predicted a better response. This finding reflects a better neuronal plasticity profile [17].

Regarding schizophrenia, a proteomic signature was identified with reduced levels of leptin and proinsulin, related to an increased risk of relapse in the short term. Early insulin, leptin, and C-peptide response during the first few weeks of treatment was associated with future relapse [15].

Elevated levels of unconjugated bilirubin (UCB) were associated with greater severity of psychotic symptoms, prolonged length of hospitalization, and aggressive behaviors during relapse. This was accentuated in patients with schizoaffective disorder. In remission, a negative correlation was identified between UCB and psychomotor retardation [18].

Finally, regarding the adverse effects of antipsychotics, it was observed that lactate levels in arterial blood rose after 90 days of antipsychotic treatment. Specifically, patients who presented extrapyramidal symptoms (dystonia and Parkinsonism) had even higher values [19].

### 3.3. Neuroendocrine Biomarkers

An attenuated response to the dexamethasone suppression test (Dex/CRH) was associated with a lower probability of remission in patients with major depression. The presence of hypercortisolemia and poor suppression of the HPA axis during the first weeks of treatment resulted in a worse clinical prognosis. However, a progressive improvement in the regulation of the shaft after the first few weeks meant the opposite [20].

In adolescents, a higher cortisol/CRP ratio in morning saliva was shown to double the risk of developing a depressive episode in the following two years. In contrast, both variables separately were not able to predict the onset of the pathology [21].

On the other hand, alterations in nocturnal secretion of growth hormone (GH) during adolescence were associated with an increased risk of developing depression and suicidal risk in adulthood. The patterns identified consisted of early GH elevation after sleep onset and persistently high levels in the early hours of the night [22].

### 3.4. Neurotrophic Factors

The studies included in this review support the neurotrophic hypothesis, showing that alteration of brain-derived neurotrophic factor (BDNF) has been associated with the pathophysiology of depression and response to antidepressant treatment. BDNF levels are lower in patients with untreated depression compared to healthy controls. Similarly, higher levels are related to greater clinical remission, especially in response to antidepressants (especially SSRIs) [23].

Another study noted that an early elevation of BDNF during the first 2 weeks of antidepressant treatment is associated with a higher likelihood of remission at 8 weeks, regardless of immediate clinical improvement. Early improvement in executive functions (inhibitory control and cognitive flexibility) was also found to be positively associated with remission [24].

### 3.5. Genetic and Epigenetic Biomarkers

In depression, a combined predictive model of clinical biomarkers, mRNA (STMN1 and PPP1R9B), and microRNA (miR-3688 and miR-5695) expression was identified, capable of predicting with great accuracy the worsening of suicidal ideation during treatment with duloxetine [25].

Regarding bipolar disorder, one study identified four SNPs on chromosome 21 (rs79663003, rs78015114, rs74795342, and rs75222709) related to the response to lithium and located in lncRNAs AL157359.3 and AL157359.4. Similarly, in an independent cohort, it was found that carriers of these alleles had a lower risk of relapse at two years [26].

In schizophrenia, a GWAS was performed in which five genetic loci were found in relation to the general response to antipsychotics (MEGF10, SLC1A, PCDH7, CNTNAP5, and TNIK). In parallel, specific associations were identified between specific SNPs and certain drugs, such as rs2239063 in CACNA1C with response to olanzapine, rs16921385 in SLC1A1 with risperidone, and rs17022006 in CNTN4 with aripiprazole [27].

Also, miRNAs showed predictive power in antipsychotic treatment. Levels of miR-30e, miR-181b, miR-34a, miR-346, and miR-7 were found to be significantly increased in patients with schizophrenia prior to treatment, and after six weeks, levels of miR-132 and miR-432 were markedly reduced in patients who improved clinically [28].

Finally, in generalized anxiety disorder (GAD), it was found that the combination of haplotype 5-HTTLPR/rs25531 (SLC6A4) and SNP rs7997012 (HTR2A) was able to modulate the response to venlafaxine [29].

### 3.6. Neuroimmaging

#### 3.6.1. Functional Connectivity (fMRI)

In young people with depression, weaker inhibitory modulation between the ventrolateral prefrontal cortex (vlPFC) and the amygdala during emotional regulation tasks was associated with greater symptom severity [30]. On the other hand, reduced excitatory connectivity between the ventromedial prefrontal cortex (vmPFC) and the amygdala after treatment with CBT and fluoxetine was associated with a higher probability of clinical remission. This means that lower prefrontal-limbic connectivity may be associated with better emotional regulation or a higher likelihood of remission [31].

In line with these findings, another study identified that greater connectivity between the amygdala and the insula was associated with a worse therapeutic response. However, in contrast to the previous paragraph, it was also found that lower baseline prefrontal-limbic connectivity was associated with a lower probability of remission. These two described patterns differentially predicted response to CBT or pharmacotherapy [13].

On the other hand, deactivation of the primary somatosensory cortex during dysphoric mood induction tasks was associated with a higher risk of long-term relapse, while lower activity in the left LPFC after treatment was associated with a lower risk of relapse [31].

In schizophrenia, 91 functional connections of the striatum with prognostic potential were identified by resting fMRI. Greater connectivity in posterior regions was associated with better clinical outcomes, as opposed to lower connectivity in frontal regions, which was associated with a better response to antipsychotic treatment. Similarly, an increased striatal connectivity index was correlated with longer duration of hospitalization in acute psychosis [32].

Finally, another study using resting fMRI identified two brain biotypes in patients with schizophrenia, based on differences in cognitive, social, and symptom performance, without depending on the DSM diagnosis. The pattern that was altered pointed to regions associated with mentalization and the network of mirror neurons [33].

#### 3.6.2. Brain Morphometry and Structure (sMRI and DTI)

Lower fractional anisotropy (FA) in white matter tracts (anterior cingulate and stria terminalis) predicted remission by up to 64% before antidepressant treatment, increasing to 74% when adding age as a moderating variable [34].

The application of machine learning (VMS) on structural magnetic resonance imaging (sMRI) is useful in classifying response to antidepressant treatment. It allowed patients to be differentiated into refractory and non-refractory with great precision. White matter was more predictive in non-refractory patients (84.65%), while gray matter was more predictive in refractory patients (67.39%) [35].

In melancholic depression, it was found by sMRI that increased CSF volume in the left Sylvian fissure was associated with poorer response to treatment and longer time to remission. Similarly, it was found that the increase in global cortical volume of CSF was correlated with a 7.8-fold increased risk of relapse or recurrence during the two years of follow-up [36].

Moving on to juvenile bipolar disorder, brain morphometry was used before and after one week of treatment with lithium and quetiapine. The baseline structural characteristics and their early changes demonstrated great accuracy in predicting the response (75%), which increased for both quetiapine (83.2%) and lithium (83.5%) when both moments were combined. In fact, cortical thickness and surface area were used for quetiapine, while hippocampal volume and cortical area were used for lithium [37].

#### 3.6.3. Receptors and Functional Neuroimaging (SPECT)

SPECT with 123I-IBZM and striatal/occipital (S/O) ratio were used to study patients with previously untreated schizophrenia by measuring the density of D2 dopaminergic receptors (D2R) in the striatum. It was found that a higher density of D2R in the striatum, as well as a higher S/O ratio were associated with worse clinical outcomes. In parallel, this higher striatal dopaminergic density was related to a worse premorbid fit (assessed by the PAS scale) [38].

### 3.7. Electroencephalography (EEG, ERPs)

Quantitative electroencephalography (qEEG) was applied to assess the brain activity of patients with depression in the early stages of treatment. An early reduction in theta concordance in the middle and right frontal region was found to correlate with a higher likelihood of remission at one week [39].

In patients with schizophrenia in remission and treated with antipsychotics, visual evoked potentials (ERPs) were used during discrimination tasks. Significantly higher latency in the NA component, as well as alterations in the N2 and P3 components, were associated with a higher likelihood of relapse in the following two years. The delay in NA latency had a sensitivity of 90% [40].

## 4. Discussion

**Figure 2:**
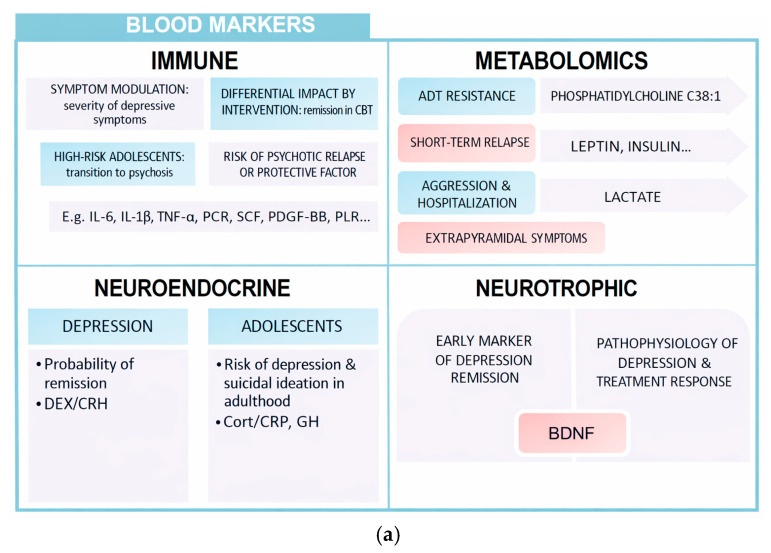

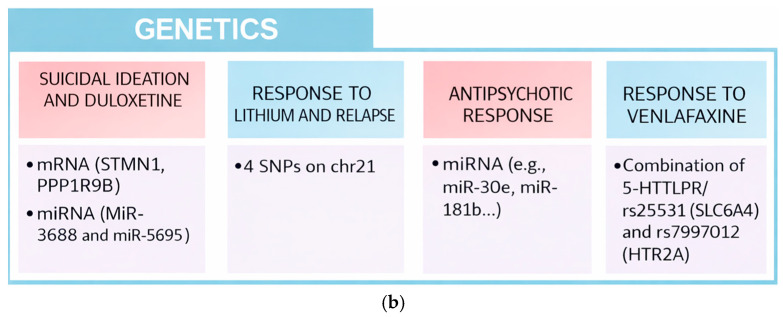
(**a**) Blood markers. (**b**) Genetic markers.

**Figure 3:**
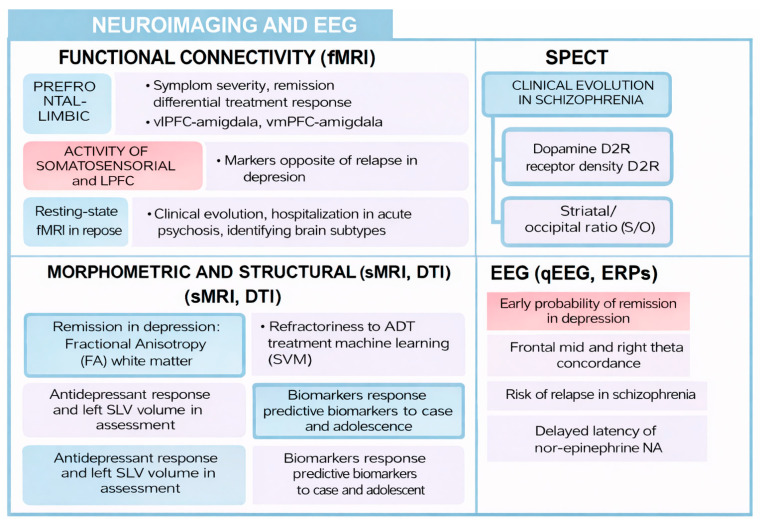
Neuroimaging and EEG biomarkers.

This review has collected different types of biomarkers with potential prognostic capacity in psychiatry (Figure 2 and Figure 3). Its integrated analysis has made it possible to identify which biomarkers are related to remission, relapse, or therapeutic response in various disorders. Next, its usefulness, methodological soundness, and real clinical implications in the context of precision psychiatry are discussed.

Peripheral markers allow an approximation to the biological substrate of many mental illnesses, with high prognostic value. This summary table shows some of the most important aspects that have been the subject of this review: immune, metabolomic, neuroendocrine, neurotrophic, and genetic.

Both modern neuroimaging techniques and electroencephalography are indirect methods of visualizing brain structure and functioning. In this review, several markers have been discussed that could have a role in certain prognostic variables associated with highly prevalent mental disorders.

### 4.1. Interpretation of the Results

The biomarkers collected in this review should not be understood as universal and stable markers, but as objective and dynamic indicators that acquire predictive value depending on the clinical and therapeutic context. In other words, they do not generally correspond to absolute values; they must be interpreted according to the relationship with the type of intervention we use, the time of the natural history of the disorder, and, of course, the patient’s profile.

Inflammatory biomarkers such as IL-6, IL-1β, TNF-α, and CRP emerged as symptomatic modulators of depression, not as indicators of global response to treatment or relapse. They were related to symptomatic severity in somatic and neurovegetative dimensions and subjective cognitive impairment. This means that they should be understood as modulators of the clinical profile [11,12]. Similarly, their ability to predict remission was closely related to the type of intervention provided, which was higher in patients treated with CBT. This reinforces the differential and contextual impact of these biomarkers [13].

Also worthy of mention in this subtype of biomarkers are the increased counts of monocytes and basophils at risk of relapse into schizophrenia. The relationship with monocytes is consistent with previous studies on the activation of microglia and dysfunction of the immune system in psychosis, while that of basophils is novel. Moreover, PLR as a protective factor implies that the balance between innate and adaptive immunity would regulate the risk of relapse [16].

Regarding metabolomic biomarkers, an interesting debate has been opened. The fact that phosphatidylcholine C38:1 is associated with a worse antidepressant response suggests that the patient’s baseline biochemistry tells us how he or she will respond. This would therefore imply that resistance to treatment would be present in the lipid profile. On the other hand, the relationship between sphingomyelin ratios and the antidepressant response reinforces the idea that neuronal plasticity is clearly linked to brain lipid metabolism [17].

We must not forget hormonal markers and their potential as predictors of relapse in schizophrenia. Leptin, C-peptide, proinsulin, and insulin are altered early during the first weeks of treatment, showing predictive potential to warn us of decompensation before the mind [15]. Furthermore, the relationship of UCB with aggressiveness and prolonged hospitalization in psychosis indicates structural vulnerability (especially in the schizoaffective phenotype) and is not an evolutionary marker [18]. Finally, regarding the adverse effects of antipsychotics, the increased lactate levels in patients who develop dystonia or Parkinsonism underline the importance of peripheral mitochondrial metabolism. This is because it could alert us to neurological toxicity before it manifests itself clinically [19].

When talking about neuroendocrine markers, we must keep in mind that the HHA axis not only tells us about stress but also about physiological rigidity. This means that a neuroendocrine system that does not react regardless of the treatment we provide or the environment is associated with a worse prognosis in depression [20]. If we add to this that the cortisol/CRP ratio in adolescents was not able to predict separately, it indicates that the problem is not at a single level. In other words, there is a loss of synchrony between systems (in this case, neuroendocrine and immune) [21]. Finally, the alteration of nocturnal GH secretion in adolescents suggests that circadian rhythms at this stage would be a useful marker of future vulnerability [22].

Moving on to neurotrophic biomarkers, BDNF again demonstrates that it is not a state marker but rather reflects global plasticity. It would indicate a worse adaptation of the nervous system of depressive patients and, by extension, guide which patients will respond and respond to treatment [23]. At the same time, this biomarker shows potential as a bridge between affective and cognitive symptomatology. That is, given its role in neurogenesis and memory, it could be linked to the improvement of cognitive functions [24].

Genetic biomarkers should be understood as predictors of sensitivity, i.e., they are useful for identifying which patients are more vulnerable to the outside and their likelihood of responding to treatment (e.g., polymorphisms or miRNAs related to response to antipsychotics) [27,28]. Above all, in this category, the importance lies in the gene-environment interaction and in how genes are expressed through epigenetics, since they are genes responsible for neuroplasticity, inflammation, and emotional response. In conclusion, it is not enough to look for a single depression gene but to integrate it into multivariate models that integrate the clinical phenotype [25,26,29]. Similarly, we must also highlight the preliminary usefulness they have shown to anticipate the response to various antidepressant and antipsychotic drugs, laying the foundations of pharmacogenetics. Functional connectivity studies indicate that there are alterations in the cross-communication of a set of regions involved in various brain processes, identifying failures in executive, perceptual, and affective circuits. However, discordance about the probability of remission and prefrontal–limbic connectivity could indicate that they are indeed context-dependent dynamic biomarkers [13,30,31]. Similarly, they are biomarkers that show potential in the prediction of the therapeutic response with differential impact, as we observed in immune markers (CBT and pharmacotherapy) [13]. Somatosensory and lateral prefrontal cortex (LPFC) deactivation are considered to be opposite predictors of depression relapse. In other words, while the former was associated with greater vulnerability in the long term, the latter paradoxically did so with a lower probability of relapse. This indicates that patients regulate their emotions in a more automatic and less forced way. Not only is it important which areas are activated, but also how much energy is used [31].

In schizophrenia, the use of resting fMRI is useful in anticipating response and clinical evolution. Specifically, the connectivity of the differential striatum with posterior and frontal regions could indicate that the more disconnected from frontal control it is, the more effective the drugs are. This could be interpreted as meaning that in these patients, the disorder would be more focused on the classic dopaminergic pathway and, therefore, they are more sensitive to the blockade of antipsychotics. In parallel, an increased global index of striatal connectivity suggests greater functional disorganization in acute phases, thus being related to the duration of hospitalization [32]. Finally, resting fMRI also allows differentiating two biotypes based on connectivity in mentalization networks and mirror neuron systems (cognitive differences, social functionality, and symptomatic load), contributing to patient stratification [33].

Finally, to elaborate on the neuroimaging and electrophysiological biomarkers, the included study using SPECT shows potential to indirectly analyze dopaminergic pathways in naïve schizophrenia. This means that a higher density of D2 receptors or S/O ratio would help us to anticipate a worse clinical outcome [38]. On the other hand, the most relevant thing about EEG is that it reflects emotional reactivity and not only basal activation, which gives it great potential for the evaluation of the antidepressant therapeutic response and the personalization of strategies at an early stage [39]. In addition, the delay of NA latency in schizophrenia showed great sensitivity as an independent predictor of relapse, as it was related to an alteration in early perceptual processing [40].

### 4.2. Limitations of the Included Studies

Despite the great potential shown in the previous section, the studies collected in this review have important methodological limitations that restrict their current clinical applicability. The small sample size in a large part of the studies is particularly noteworthy, especially in those of functional connectivity and some inflammatory studies.

If we focus on studies focused on machine learning, for example, we find that many of these analyses are based on small samples and limited validation strategies. So, these findings should be framed as exploratory or proof-of-concept rather than as evidence of robust predictive biomarkers. Greater reproducibility and external validation of these findings are therefore needed. Another limitation to note is the low level of external replication in many studies that propose predictive models using machine learning or multivariate analysis. Methodological heterogeneity is also a problem, as well as the use of different types of neuroimaging tests, analytical protocols, clinical classifications, statistical thresholds, and inclusion criteria. This makes it difficult to compare between studies and draw conclusions. Publication bias is also relevant, as few studies report negative results or apply corrections for multiple comparisons. Finally, in some subtypes of biomarkers (such as metabolomic or structural), the observational design and exploratory nature of the studies do not allow causal relationships to be established. In conclusion, these discrepancies explain the current low integration of biomarkers in healthcare psychiatry. This means that, despite the promising advances of this new biological psychiatry, the existence of clinical decision rules based on biomarkers that can be generalized to a multitude of different disorders may be a reality that is, in fact, not as close to being achieved as we would like.

### 4.3. Strengths and Limitations of the Review

This review presents a series of strengths that support the validity of the findings described. Firstly, it is comprehensive and compiles different types of blood, genetic, neuroimaging, and electrophysiological biomarkers in an integrative manner, encompassing different methodologies and prognostic perspectives. This has allowed a comparative interpretation to be made between clinical dimensions and biological systems involved. In addition, both the adult, child, and adolescent populations have been contemplated with the aim of increasing representativeness. The clinical perspective, focused on the prognostic value (clinical evolution, response to treatment, and relapse), favors applicability to real clinical practice.

Another relevant strength is that the methodology has followed a logical sequence of search and selection of studies, which has ensured a transparent and replicable structure. Clear inclusion and exclusion criteria were defined, a methodical screening process was carried out, and the findings were organized according to biomarker subtype. The selection of final studies is rigorous, as it includes only original studies and excludes other reviews, meta-analyses, and preclinical studies. Regarding the table of results, it is extensive and complete, collecting key information from each study and validated in each entry against the original article. Similarly, a meticulous assessment of the level of evidence and the risk of bias of the studies has been carried out, using standardized tools adapted to each design (RoB 2, NOS, AXIS).

However, this review is not without limitations. The main one is that it is not a systematic review in the strict sense, as peer screening has not been carried out, and there is no search protocol registered. This may lead to some selection biases that limit the validity of the conclusions. Similarly, despite various psychiatric disorders, there is a clear predominance of depression and schizophrenia, leaving aside other disorders. Something similar occurs with the predominance of studies focused on adults, with the child and adolescent population being underrepresented. Likewise, the articles included were limited to the English language.

On the other hand, the possibility of generalizing results has been limited due to the great clinical and methodological heterogeneity between studies. For this reason, this is a qualitative and descriptive review, without joint statistical analysis (meta-analysis). Despite having carried out an evaluation of methodological quality, the design of some of the studies is observational and exploratory, with little replication and decreased sample sizes. In addition to all this, there is a significant dispersion of results (especially in functional connectivity and inflammation) that limits the extraction of consistent results.

### 4.4. Clinical Implications and Future Research

Biomarkers show great potential to be the next step in the evolution of a more individualized, objective, and biology-based psychiatry. This review has highlighted its ability to predict clinical evolution, therapeutic response, and risk of relapse. However, their actual applicability remains limited, and the integration of biomarkers into healthcare practice remains in the preliminary phase.

Currently, its most reasonable clinical value is not diagnostic but prognostic and stratifying. Its use would make it possible to personalize prevention and follow-up strategies, with the aim of identifying patients at greater risk of relapse or chronification. In this sense, the distinction between static (morphometric or genetic) and dynamic (inflammatory, EEG, or functional connectivity) biomarkers will be of great importance in devising tools adapted to the different phases of the clinical course.

Future research should be directed towards the design of multimodal predictive models that integrate biomarker subtypes. This convergence will allow us to reflect the biological complexity of psychiatric disorders and design clinically useful predictors. Therefore, the development of these models must be supported by artificial intelligence capable of integrating heterogeneous data and guiding clinical decision-making. Similarly, adopting transdiagnostic approaches that go beyond traditional categories would allow us to identify common vulnerability mechanisms and profiles.

By delving into each subtype of biomarker and its specific applications, inflammatory biomarkers allow the identification of clinical profiles with somatic or neurovegetative symptoms or subjective cognitive impairment. Its most promising application would be as modulators of clinical phenotype and as intervention guides (e.g., use of CBT in proinflammatory profiles). Metabolomic biomarkers reflect the patient’s baseline biochemistry, which is useful for predicting therapeutic resistance or vulnerability to adverse effects. Neuroendocrine biomarkers inform us about the reactivity of the HPA axis and its interaction with other systems (e.g., immune), indicating physiological styles of adaptation and vulnerability to stress. As for neurotrophic drugs, they allow us to analyze brain plasticity and can be used to monitor the biological response to treatment.

Functional connectivity shows great potential to more accurately predict therapeutic response and risk of relapse and to define transdiagnostic functional biotypes for the individualization of treatments. Structural neuroimaging acts as a trait biomarker, helping to identify resistance, chronicity, or low plasticity. On the other hand, SPECT allows us to evaluate dopaminergic activity in vivo and apply it in the stratification of subtypes sensitive to antipsychotics. Finally, EEG, being so accessible and sensitive to change, shows potential to be integrated as a monitoring and therapeutic anticipation tool, especially in the early stages.

The gradual introduction of these biomarkers in the coming years will not only depend on technical advances but also on the ability of the system to guarantee their accessibility, standardization, and ethical use. Biomarkers will be really useful in diverse healthcare settings, not only in highly specialized environments. This implies moving towards cost-effective techniques, homogeneous protocols, and common interpretative criteria. Above all, its incorporation must prioritize equity in access, data protection, and the ethical impact of the use of biomarkers in mental health.

## 5. Conclusions

This work highlights the potential of biomarkers as complementary tools in psychiatry, with specific applications in the prediction of the clinical course, therapeutic response, and risk of relapse. Far from replacing the clinic, these tools should be understood as a natural extension of psychiatry. In this sense, they do not come to resolve the debate between the objective and the subjective but to offer one more way of approaching the complexity of the human being.

Throughout this review, different subtypes of biomarkers have been explored, and consistent patterns have been identified. Although the healthcare integration of these indicators is still limited, this review shows that there are already solid findings that expand the path of a more precise and less reductionist psychiatry. However, the results obtained should be interpreted with caution since the included studies have some relevant limitations, such as small sample size, heterogeneity of designs, low external replication, or publication bias.

Looking ahead, the development of validated and replicable multimodal predictive models that relate biological data to clinical trajectories is needed. This will involve incorporating contextual variables such as the psychopathological profile, the therapeutic history, and the psychosocial environment of the patient. Similarly, it will be necessary to implement criteria of clinical applicability and equitable accessibility to guarantee its ethical and effective use. The standardization of protocols, the training of health personnel, and the design of affordable and interpretable tools will be essential steps for their gradual implementation.

The future of psychiatry could lie in overcoming the dichotomy of the biological and psychosocial [41]. Biomarkers do not represent a biological dogma but an integrating possibility by recognizing the biological uniqueness of each patient and, at the same time, their human experience. A truly integrative psychiatry will be able to combine empathic listening with biological reading. So, biomarkers are an inevitable step in the progress of psychiatry towards a more humane, precise, and fair medicine.

## Figures and Tables

**Table 1:** Revised studies.

Author and Year	Study Design	Population	Biomarkers Evaluated	Evaluation Method	Level of Evidence	Risk of Bias	Main Findings
Kofod et al., 2022 [11]	Longitudinal observational study.	90 outpatients with moderate to severe depression (71% women, mean age 38 years)	27 inflammatory markers, including IL-6, TNF-α, CRP, IL-9, IL-12, and IL-15.	Blood tests at different time points (26 weeks) during treatment with escitalopram or nortriptyline. Symptoms were assessed using the MADRS scale.	Moderate, although not an RCT, it has good methodological quality, longitudinal follow-up, and extensive biomarker analysis.	Moderate, as it is a prospective design, with a standardized collection of biological samples and validated scales. Even so, the absence of a control group limits part of the causal interpretation.	No relationship between baseline levels or changes in inflammatory markers and overall response to treatment or long-term risk of relapse. Yes, correlation between baseline inflammatory markers and severity of specific symptoms of depression (somatic, neurovegetative, and affective).
Wang et al., 2023 [12]	Prospective observational study.	114 patients with major depressive disorder (MDD)	48 plasma immune cytokines, including basic FGF, IFN-γ, IL-1β, MCP-1, M-CSF, SCF, IL-9, RANTES, and PDGF-BB.	Luminex assay for cytokine measurement, PDQ-D questionnaire for subjective cognitive function, and HDRS-17 for depressive symptoms.	High, longitudinal design with a significant sample size and robust methodology.	Low; objective method plus double evaluation with PDQ-D and HDRS.	Elevated levels of basic FGF, IFN-γ, IL-1β, MCP-1, M-CSF, and SCF and reduced levels of IL-9, RANTES, and PDGF-BB were associated with subjective cognitive impairment. A multivariable logistic model based on SCF and PDGF-BB combined with baseline PDQ-D scores showed high accuracy in predicting this residual cognitive decline after treatment.
Dunlop et al., 2017 [13]	Randomized clinical trial (PReDICT study).	344 adults (18–65 years) with major depressive disorder (MDD), without previous treatment for current depression, and with moderate-to-severe symptoms	Inflammatory cytokines: IL-6, IL-1β, TNF-α, CRP. Dex/CRH neuroendocrine test. DNA polymorphisms and mRNA expression. Resting functional resonance imaging (fMRI) and DTI.	Blood tests for cytokines. Genetic evaluation by DNA extraction and mRNA expression. Brain imaging with fMRI and DTI before and after treatment. Dex/CRH test (pre and post treatment). Psychological and personality evaluation at the beginning. Randomization with escitalopram, duloxetine, or CBT for 12 weeks. Follow-up for 21 months.	High, randomized clinical trial, robust design with multiple biological measures and longitudinal follow-up.	Low; randomized design, use of standardized interventions, control for confounding variables, and longitudinal evaluation reinforce internal validity. Prior registration was carried out.	Elevated IL-6 levels and CRP at baseline were associated with lower remission in patients treated with CBT, but not with drugs. Increased amygdala-insula connectivity predicted worse response to treatment. Lower baseline prefrontal-limbic connectivity was associated with a lower likelihood of remission. These patterns allowed for discriminating between responders to CBT or pharmacotherapy. The Dex/CRH test had no isolated predictive value but was included in the combined model.
Mongan et al., 2020 [14]	A prospective multicenter study nested in two cohorts (EU-GEI and ALSPAC) with classification analysis supervised by machine learning (Support Vector Machine).	133 individuals in a state of high clinical risk (EU-GEI) for psychosis selected from mental health services and 70 adolescents without psychotic experiences at 12 years of age, followed up to 18 years of age; follow-up was 24 months	Plasma proteins related to psychotic transition, including Alpha-2-macroglobulin (A2M), IGHM, C4BPA, C8A, and PLTP.	Proteomic analysis of basal plasma samples by mass spectrometry. Predictive model with machine learning algorithms.	High, multicenter longitudinal design, objective techniques, adequate sample size, and cross-validation of the model.	Moderate; potential risk in cohort heterogeneity, but minimized by clear inclusion criteria and robust statistical analysis.	Protein panels were identified that predicted the transition to psychotic disorder in high-risk individuals and the onset of psychotic symptoms in adolescents. Predictive models showed moderate accuracy and clinical utility for early interventions. The most involved pathways were complement activation and the coagulation cascade.
Schwarz et al., 2012 [15]	Prospective longitudinal study.	77 patients with schizophrenia (mean age 38 years) without antipsychotic treatment at baseline	Panel of 181 serum biomarkers, including cytokines, growth factors, and hormones. MMP-7, CCL20, IL-16, and VEGF stood out.	Multiplex immunoassay (xMAP) in blood serum and clinical follow-up for 25 months with PANSS and CGI scales.	High, well-designed longitudinal study with advanced proteomic tools and prolonged follow-up.	Moderate; potential risk in the lack of control of confounding factors such as intercurrent treatments and uncontrolled biological variability.	Identification of proteomic profiles associated with three prognostic domains: clinical severity, early response to treatment (6 weeks), and time to relapse. The combinations of biomarkers allowed the construction of predictive models with AUCs greater than 0.85, partially replicated in independent cohorts.
Llorca-Bofí et al., 2023 [16]	Longitudinal study.	111 patients with schizophrenia in remission after a first psychotic episode (PEF)	Inflammatory cell count: monocytes, eosinophils, lymphocytes, PLR, and BLR ratios.	Blood tests in remission. Clinical evaluation for 3 years to detect relapse. Multivariate analysis and ROC curves.	High, large cohort with prolonged follow-up, robust statistical analysis, and confounding control.	Moderate; without an external control group or validation in other samples but adjusted for multiple comparisons and factors.	Elevated monocyte and basophil count at baseline was associated with increased risk of psychotic relapse. Basophil count remained an independent predictor after adjusting for clinical variables and substance use. However, a high ORP value was a protective factor. The optimal cut-off values to predict relapse were monocytes > 0.52 × 10^9^/L (AUC = 0.66) and basophils > 0.025 × 10^9^/L (AUC = 0.75).
Czysz et al., 2019 [17]	Prospective observational study.	159 patients with major depressive disorder (MDD) on antidepressant treatment	Plasma metabolites: sphingomyelins, lysophosphatidylcholines, phosphatidylcholines, and acylcarnitines.	Targeted metabolomics using the AbsoluteIDQ p180 kit.	Moderate, prospective design with standardized follow-up, adequate sample size, and validated analytical tool (recognized metabolomics platform). Although exploratory metabolomic analysis.	Low; systematized procedure, no conflicts of interest, robust statistical analyses.	High concentrations of phosphatidylcholine C38:1 at baseline were associated with less improvement in depressive symptoms after treatment. On the other hand, a higher proportion of hydroxylated sphingomyelins compared to non-hydroxylated sphingomyelins was related to a better clinical response.
Gama Marques et al., 2020 [18]	Comparative longitudinal study.	88 with schizophrenia or schizoaffective disorder, compared to 44 bipolar patients. Follow-up for 12 months	Plasma levels of unconjugated bilirubin (UCB).	Blood tests of bilirubin in the relapse and remission phases. Psychopathological evaluation using PANSS, CGI, and PSP.	Moderate, longitudinal design, repeated clinical and biological measures, correlational analysis, but no healthy control group or external replication.	Moderate; good control of medical comorbidities and substances, but no randomization or follow-up.	Higher levels of UCB were associated with the relapse phase in patients with schizophrenia and schizoaffective disorder. In those with schizoaffective disorder, it was positively associated with symptom severity and length of hospitalization. A positive correlation was found between aggressive and negative behaviors with psychomotor retardation in the remission phase.
Elmorsy et al., 2016 [19]	Prospective longitudinal study.	88 patients with schizophrenia or bipolar disorder, treated with typical or atypical antipsychotics. Follow-up for 90 days	Blood lactate levels.	Arterial lactate tests at baseline, on the tenth day, and at 90 days of treatment. Clinical evaluation of extrapyramidal symptoms using the mAIMS scale.	Moderate, longitudinal follow-up with objective measures, but no healthy control group or randomization.	Moderate; without blinding or randomization, but with control for comorbidities and repeated biological measures.	Lactate levels rose significantly after antipsychotic treatment, especially with haloperidol and chlorpromazine. Elevated levels were associated with an increased risk of extrapyramidal symptoms (dystonia and Parkinsonism).
Ising et al., 2007 [20]	Prospective longitudinal study.	50 patients with major depression were admitted to the psychiatric hospital unit (mean age 44), and 40 healthy controls	Activity of the HPA axis by Dex/CRH neuroendocrine test.	DEX/CRH test performed twice, in the first 48 h after admission and 15–21 days after the start of treatment. They defined axis normalization as a reduction in peak cortisol >50 ng/mL between the first and second tests.	Moderate, robust longitudinal design, but no randomization or placebo group. Well-validated techniques and clear clinical follow-up.	Low; consecutive inclusion, standardized diagnostic criteria, objective results, and detailed statistical analysis.	36 of 50 patients showed endocrine improvement of the HPA axis after treatment. 64% of clinical responders at 5 weeks, 90% clinical referral at discharge. Patients without endocrine improvement had a 4.42-fold increased risk of non-response.
Landau et al., 2021 [21]	Longitudinal randomized clinical trial.	122 adolescents at risk of depression (12 to 16 years old)	Morning salivary cortisol, salivary CRP, and Cort/CRP ratio.	Salivary measurements were collected in duplicate during two consecutive days at three times (baseline, post-intervention, and at two years). Morning and evening ratios were calculated, as well as daytime slopes.	High, longitudinal, and randomized prospective design. Rigorous and duplicate evaluation of biomarkers. Multivariate analysis adjusted for multiple covariates of relevance.	Low; adequate control of confounding factors. A well-characterized sample with a high percentage of complete follow-up. Robust analysis with multiple imputation and statistical validation of assumptions.	A high Cort/CPR ratio significantly predicted the onset of a new depressive disorder in the following two years. On the other hand, neither cortisol nor CRP separately showed predictive value. The effect was maintained by adjusting for age, sex, BMI, socioeconomic status, history of anxiety, sleep, and previous depressive symptoms.
Coplan et al., 2000 [22]	Longitudinal comparative study.	77 adolescents (with and without a diagnosis of major depression) were followed up into adulthood	Nocturnal secretion of growth hormone (GH).	GH sampling every 20 min for 24 h, coinciding with the third night of polysomnography.	Moderate, the study was longitudinal, which provides prognostic value, with follow-up until adulthood. However, the sample size is limited and was not replicated in other cohorts.	Moderate; despite being a robust design, the sample is small, and there is no clear mention of control measures and randomization.	Nocturnal GH secretion in adolescence predicts depressive episodes and suicidal behaviors in adulthood. Individuals who had a greater secretion in the first 4 h after the onset of sleep had a higher risk of suicide. In contrast, adults with a history of major depression had less discharge before sleep onset during their adolescence.
Molendijk et al., 2011 [23]	Naturalistic longitudinal study.	77 patients with major depression, without antidepressant treatment at the beginning. Follow-up for 8 weeks of SSRI treatment	Serum levels of brain-derived neurotrophic factor (BDNF). Executive functions (TMT, Stroop…).	Plasma levels of BDNF. Neuropsychological battery at baseline, week 2, and week 8.	Moderate, longitudinal design with multiple assessment points and objective measures. However, there was no healthy control group.	Moderate; possible selection bias (outpatient consultation population), and no blinding is mentioned. However, standardized measures and robust analysis.	Early abnormalities in BDNF (especially in the first two weeks) were associated with a higher likelihood of remission at week 8. This correlation was independent of early symptomatic improvement (suggesting prognostic value of the individual). Early improvement in executive functions was also associated with remission (especially inhibitory control and cognitive flexibility).
Tadić et al., 2011 [24]	A prospective longitudinal study was included in the randomized clinical EMC trial (Early Medication Change vs. Treatment As Usual).	40 patients with major depressive disorder in a current episode (20 with remission after two weeks and 20 without early remission)	BDNF and executive functions.	Serum BDNF levels by ELISA and neuropsychological battery.	High, well-defined sample, longitudinal, objective measurement, and multivariate analysis.	Moderate. Good methodology and control, but a relatively small sample to generalize, and no external replication.	The group with early remission at 2 weeks showed a greater elevation of BDNF levels and better performance in executive functions. Both BDNF and cognitive performance were independent predictors of remission at 8 weeks.
Belzeaux et al., 2019 [25]	Randomized clinical trial.	237 patients with major depressive disorder treated with duloxetine (*n* = 112) or placebo (*n* = 125) for 6–8 weeks	Peripheral expression of mRNA (PPP1R9B, STMN1) and microRNA (miR-3688, miR-5695).	Blood gene expression analysis before treatment and clinical follow-up of suicidal ideation (item 10 MADRS) up to 8 weeks.	High, robust experimental design, use of multivariate models, and statistical validation of the predictive model.	Low; randomization, placebo group, objective measures, and precision measures (AUC = 0.94 for the combined model).	Levels of STMN1 and miR-5695 predicted worsening of suicidal ideation during duloxetine treatment. The combined clinical and molecular model showed high predictive capacity (AUC = 0.94).
Hou et al., 2016 [26]	Genome-wide association study (GWAS).	2563 patients with bipolar disorder treated with lithium, collected from 22 ConLiGen participating sites	Genome-wide single-nucleotide polymorphisms (SNPs).	Genotyping and statistical analysis to identify SNPs related to lithium response. Alda scale to measure response to treatment.	High, large sample size and multicenter design that increase statistical power and extrapolation of results. Validation of findings in an independent cohort.	Low. Use of a standardized scale (Alda) to assess response to treatment. Rigorous statistical analysis and control of possible confounding factors.	Identification of four SNPs on chromosome 21 significantly associated with lithium response (rs79663003, rs78015114, rs74795342, and rs75222709). In an independent prospective study of 73 patients treated with lithium for 2 years, carriers of the alleles associated with the response had a significantly lower relapse rate compared to carriers of alternative alleles. On the other hand, the associated region contains two genes that encode long non-coding RNAs (lncRNAs), AL157359.3 and AL157359.4. They could play a role in regulating gene expression in the central nervous system.
Yu et al., 2018 [27]	Genome-wide association study (GWAS).	2012 patients with schizophrenia treated with antipsychotics and a follow-up of 6 weeks	SNPs related to genes MEGF10, SLC1A1, PCDH7, CNTNAP5, TNIK, CACNA1C, CNTN4.	Genotyping and analysis in independent cohorts. Functional validation of some SNPs by gene expression and *pathway analysis*. Evaluation of the response with PANSS and CGI scales.	High, large sample, standardized genetic methodology, subgroup analysis by type of antipsychotic, and partial replication.	Low; large sample, validated tools, stratified analysis, good definition of response phenotypes, and control of multiple confounding factors.	5 genetic loci related to the general response to antipsychotics were identified. Specific associations were found between SNPs and specific drugs (risperidone, olanzapine, aripiprazole…).
Sun et al., 2015 [28]	Case–control study with longitudinal follow-up.	61 patients with schizophrenia, 62 healthy controls, and a subgroup of 25 patients followed for 6 weeks of treatment	MicroARN plasmatic miR-30e, miR-181b, miR-34a, miR-346, miR-7, miR-132, miR-432, miR-212.	Expression analysis by qPCR in peripheral blood before and after treatment. Clinical evaluation with PANSS, CGI, and GAS.	Moderate, well-described molecular analysis and clinical follow-up, but a small sample in the longitudinal subgroup.	Moderate; no mention of randomization, small longitudinal subgroup size. Yes, good clinical correlation and sensitivity and specificity analysis.	A panel of microRNAs composed of miR-30e, miR-181b, miR-34a, miR-346, and miR-7 presented high diagnostic value. After treatment, levels of miR-132, miR-181b, miR-30e, and miR-432 decreased significantly, associated with clinical improvement. MiR-132 and miR-432 were reduced in those patients with the best antipsychotic response.
Lohoff et al., 2010 [29]	Retrospective genetic study (pharmacogenetic).	156 patients with generalized anxiety disorder (GAD) treated with venlafaxine XR for 12 weeks	SNPs 5-HTTLPR/rs25531 (in gene SLC6A4) and rs7997012 (in gene HTR2A).	Genotyping by PCR and SNP analysis. Clinical evaluation by the Hamilton scale of anxiety (HAM-A).	Moderate, limited sample, but specific analysis, with systematic evaluation of clinical response and cross-validation of gene-gene interactions.	Moderate; no placebo group, risk of confounding due to uncontrolled clinical variables, but well-defined markers and standardized evaluation.	The combination of the HTTLPR/rs25531 and rs7997012 polymorphisms was significantly associated with a greater reduction in symptoms. A gene-gene interaction that modulated the response to treatment was described.
Kung PH et al., 2022 [30]	Secondary analysis of randomized controlled trials.	140 young people (12–25 years old) with major depression	Effective connectivity between ventral frontal cortex (vlPFC) and amygdala.	Functional magnetic resonance imaging (fMRI) during cognitive reassessment tasks.	High, experimental design with fMRI, a representative sample, and analysis based on specific tasks related to emotional regulation.	Low; controlled trial-based analysis, robust design, and low risk of bias in data collection and interpretation.	An inhibitory modulation between the ventrolateral prefrontal cortex (vlPFC) and the amygdala was associated with greater clinical severity.
Farb et al., 2022 [31]	Randomized clinical trial.	85 patients with major depression in remission, treated with CBT combined with fluoxetine or placebo	Functional connectivity of the ventromedial prefrontal cortex (vmPFC) and amygdala. Somatosensory cortex activation.	fMRI before and after cognitive therapy (for 8 weeks) or mindfulness-based therapy during mood-inducing tasks.	High, randomized design, longitudinal follow-up, use of objective techniques, and post-intervention evaluation.	Low; randomized, standardized interventions, evaluation with objective neurofunctional measures.	Lower excitation of vmPFC over the amygdala was associated with a higher likelihood of remission after treatment with CBT and fluoxetine. Somatosensory deactivation (primary somatosensory cortex) during dysphoric mood induction was associated with an increased risk of relapse at two years. Lower activation of the left LPFC after treatment was associated with a lower risk of relapse.
Sarpal et al., 2016 [32]	Prospective study.	41 patients with acute psychosis	Functional connectivity in the resting state of the striatum.	Resting-state functional magnetic resonance imaging (fMRI) and functional connectivity analysis.	High use of objective techniques, a specific clinical sample, and a longitudinal approach.	Low; robust neuroimaging analysis of patients evaluated prior to initiation of treatment.	A total of 91 functional connections were identified. Lower connectivity with frontal regions was associated with better response to antipsychotics, while greater connectivity with posterior regions was associated with better clinical response. A high striatal connectivity index was associated with longer duration of hospitalization.
Viviano et al., 2018 [33]	Cross-sectional study.	116 patients with schizophrenic spectrum disorders and 133 healthy controls	Functional connectivity in a resting state between brain networks (cognitive control, default network, and salience).	resting-state fMRI and functional connectivity analysis between neural networks.	Moderate, large sample with a control group and multivariate analysis, but a cross-sectional design limits the direct predictive value (it does not allow causal inferences or longitudinal prognosis).	Low; good methodological quality, although without temporal follow-up or replication in other cohorts. Replicated analysis in an independent sample.	Alterations in connectivity between cognitive control networks and salience were associated with worse clinical functioning. Identification of two brain biotypes differentiated by mentalization regions and the mirror network, associated with worse cognitive performance, social functioning, and greater symptomatology.
Korgaonkar et al., 2014 [34]	Prospective study.	20 patients with major depressive disorder	Fractional anisotropy in white matter tracts of the anterior cingulate and stria terminalis.	Diffusion tensor imaging (DTI) before antidepressant treatment.	Moderate, small sample, but objective method and longitudinal follow-up with specific prediction.	Moderate; robust neuroimaging technique, but limited sample size.	Altered connectivity in the anterior cingulate and stria terminalis tracts predicted remission to treatment with an accuracy of 62%, increasing to 74% when age was included in the model.
Gong et al., 2011 [35]	Prospective study with machine learning (SVM) techniques.	61 patients with major depression (without previous treatment) and 42 healthy controls	Volume of gray and white matter by brain morphometry.	Structural MRI before treatment. Classification into refractory and non-refractory patients after treatment.	Moderate, objective techniques, multivariate analysis (MVS), cross-association, and association with clinical evolution. However, the sample size was small.	Low; no randomization, but a healthy control group. Standardized method and exclusion of previous pharmacological effects.	Pre-treatment brain structural features classified patients into refractory and non-refractory. White matter was more accurate in non-refractory (84.65%) and gray matter in refractory (67.39%). The neuroanatomical pattern had differential prognostic value, since it varied according to the type of depression.
Cardoner et al., 2003 [36]	Prospective longitudinal observational study.	55 patients with major depression with melancholic features (DSM-IV), evaluated at baseline and followed for 6 months (acute phase) plus 2 years (follow-up phase)	Volume of cortical global CSF and the left Sylvian fissure.	Structural magnetic resonance imaging, morphometric analysis of CSF volume, clinical evaluation of remission (phase I), and relapse/recurrence (phase II).	High, good sample, longitudinal design, objective imaging techniques, quantitative analysis of volume, and correlation with clinical outcome.	Moderate; without a healthy control group, without randomization, but with standardized prospective evaluation and multivariate analysis.	CSF enlargement in the left fissure of Silvio was associated with a longer time to phase I remission (remission in 82 days vs. 35 days). Increased overall cortical volume was associated with a 7.8-fold increased risk of relapse or recurrence during phase II.
Lei et al., 2022 [37]	Randomized clinical trial.	121 adolescents and young adults with bipolar disorder in the phase of acute treatment with lithium or quetiapine	Brain morphometric measurements (cortical thickness, surface area, and subcortical volume).	Structural MRI before treatment and after one week. Analysis using deep learning for response prediction.	High, randomized design, prospective approach, use of structural MRI, and cross-validation of the predictive model.	Low; randomized, standardized interventions, well-defined assessment times, advanced statistical analysis, and control of clinical variables.	Pretreatment brain morphometric features and acute changes in the first week of treatment predicted response to treatment with greater than 75% accuracy. The combination of both moments improved predictive accuracy (quetiapine 83.2%, lithium 83.5%).
Pérez et al., 2003 [38]	Comparative observational study (case–control).	9 patients with previously untreated schizophrenia, classified according to good or poor prognosis criteria (DSM-IV), compared to 9 paired healthy controls	Density of the D2 dopaminergic receptor in the striatum.	SPECT with 123I-IBZM, striatal/occipital ratio (S/O) as a receptor density index.	Low, specific, and validated technique (SPECT), but small sample, exploratory nature, and preliminary results.	High; without randomization or blinding, limited size, but with well-defined groups and control of relevant clinical variables. There is no external validation.	Patients with a worse clinical prognosis had a higher density of D2R in the striatum (higher S/O). Higher density was associated with worse premorbid adjustment (SBP). An upregulation of D2R secondary to reduced prefrontal function is suggested as a negative prognostic marker in naïve schizophrenia.
Cook et al., 2009 [39]	Randomized, placebo-controlled clinical trial.	72 adults with unipolar major depressive disorder	Concordance in the theta band of the EEG in the middle and right frontal regions.	Quantitative electroencephalography (EEG) was performed in the early stages of antidepressant treatment.	High, experimental design, placebo control group, non-invasive physiological biomarker, and repeated measures.	Low; clear randomization, placebo use, objective assessment, and well-described statistical analysis.	Early decreases in theta concordance in the middle and right frontal region were associated with a higher likelihood of remission with antidepressant treatment.
Matsuoka et al., 1999 [40]	Prospective longitudinal study.	44 outpatients with schizophrenia in remission under maintenance treatment. 20 healthy controls. Follow-up for 2 years	Visual evoked potentials (ERPs), NA, N2, and P3 components.	ERP registration during the letter discrimination task. Comparison of patients with and without relapse at follow-up.	Moderate, objective method, long follow-up, clear functional differences between groups, but no external replication.	Moderate; without randomization or blinding, but good clinical control of variables and treatment.	Delayed latency of the NA component at baseline was associated with the risk of psychotic relapse in the following two years (90%).

The studies are ordered according to their order of appearance in the text. Abbreviations can be consulted at the end of the manuscript.

## Data Availability

No new data were created or analyzed in this study. Data sharing is not applicable to this article.

## Abbreviations

The following abbreviations are used in this manuscript:

AXIS	Appraisal Tool for Cross-Sectional Studies
BDNF	Brain-Derived Neurotrophic Factor
CRH	Corticotropin-Releasing Hormone
CRP	C-Reactive Protein
DSM	Diagnostic and Statistical Manual of Mental Disorders
DTI	Diffusor Tensor Imaging
EEG	Electroencephalogram
FA	Fractional Anisotropy
GH	Growth Hormone
GWAS	Genome-Wide Association Study
HPA	Hypothalamic–Pituitary–Adrenal Axis
IL	Interleukin
INF	Interferon
SSRI	Selective Serotonin Reuptake Inhibitors
CSF	Cerebrospinal Fluid
LPFC	Lateral Prefrontal Cortex
mRNA	Messenger RNA
miRNA	MicroRNA
MRI	Magnetic Resonance Imaging (fMRI: functional)
NA	Negative Afterpotential (potencial visual tardío)
NOS	Newcastle-Ottawa Scale
PLR	Platelet-to-Lymphocyte Ratio
SAF	Schizoaffective Disorder
SCF	Stem Cell Factor
SVM	Support Vector Machines
GAD	Generalized Anxiety Disorder
CBT	Cognitive-Behavioral Therapy
TNF	Tumor Necrosis Factor
UCB	Unconjugated Bilirubin
